# Cultural Differences in Perceiving Sounds Generated by Others: Self Matters

**DOI:** 10.3389/fpsyg.2015.01865

**Published:** 2015-12-02

**Authors:** Liyu Cao, Joachim Gross

**Affiliations:** School of Psychology, University of GlasgowGlasgow, UK

**Keywords:** sensory attenuation, cross cultural differences, forward model, self-construal, top–down modulation

## Abstract

Sensory consequences resulting from own movements receive different neural processing compared to externally generated sensory consequences (e.g., by a computer), leading to sensory attenuation, i.e., a reduction in perceived intensity or brain evoked responses. However, discrepant findings exist from different cultural regions about whether sensory attenuation is also present for sensory consequences generated by others. In this study, we performed a cross culture (between Chinese and British) comparison on the processing of sensory consequences (perceived loudness) from self and others compared to an external source in the auditory domain. We found a cultural difference in processing sensory consequences generated by others, with only Chinese and not British showing the sensory attenuation effect. Sensory attenuation in this case was correlated with independent self-construal scores. The sensory attenuation effect for self-generated sensory consequences was not replicated. However, a correlation with delusional ideation was observed for British. These findings are discussed with respects to mechanisms of sensory attenuation.

## Introduction

Auditory processing is influenced by top–down processes. For example, self-generated tones are perceived as lower in intensity as compared to externally generated tones (Sato, 2008; Weiss et al., 2011), known as the sensory attenuation effect (sensory attenuation for the self). Interestingly, Sato (2008) also found that merely watching others generate a tone (by pressing a button) also makes the tone be perceived lower in intensity (sensory attenuation for others). This indicates that the fact of watching/knowing a tone was generated by others’ acts as a top–down factor to modulate auditory perception. However, sensory attenuation for others was not successfully replicated later with a very similar testing paradigm (Weiss et al., 2011), noting that Sato (2008) study was from Japan and Weiss et al. (2011) study was from Germany. This raises the possibility that the effect is contingent on the cultural backgrounds of participants.

Findings from cross cultural studies indicate that easterners (including but not limited to people from Asian countries like China and Japan) have more interdependent selves and westerners (including but not limited to people from European countries like UK and Germany, and North American countries like USA) have more independent selves (Markus and Kitayama, 1991). The difference between interdependent and independent selves manifests in many aspects. Generally speaking, people with more interdependent selves give more weight to their social (interpersonal) self than their personal self, which makes the influence from others more profound. On the contrary, people with more independent selves are influenced by others to a lesser degree (Markus and Kitayama, 1991). Numerous studies have demonstrated how self-construal has a profound influence on various aspects of social cognition (Han and Northoff, 2008; Cross et al., 2011). For example, Chinese participants have less preference to their own face over others’ faces than British participants (Sui et al., 2009), and further studies showed that this difference may be modulated by self-construal priming (Sui et al., 2013). When interacting with others, people with more interdependent self-construal tended to unconsciously mimic other’s behavior (e.g., face rubbing) more than people with more independent self-construal (van Baaren et al., 2003). It was suggested that the difference in self-construal could be fundamental to cultural differences in cognition (Varnum et al., 2010). The dominance of interdependency over independency may make the differentiation between self and others less salient among easterners than among westerners. Thus we hypothesize that easterners’ perceptual experience toward a tone generated by others is similar to a self-generated tone and they show sensory attenuation for both self and others_._ While for westerners, there is a clear differentiation between self and others so that others generated tone is just like an external tone. Thus we hypothesize that there is sensory attenuation for the self but not for others among westerners.

To summarize, we predict a cultural difference in sensory attenuation for other-generated tones, with people from collectivism-dominated cultural backgrounds (e.g., Chinese) showing this effect and people from individualism-dominated cultural backgrounds (e.g., British) not showing this effect. We hypothesize that there is no cultural difference with respect to sensory attenuation for the self, which is accounted for by the common forward model mechanism (Wolpert and Ghahramani, 2000; see Discussion). Further support for this hypothesis is that sensory attenuation for the self was shown in both Sato (2008) and Weiss et al. (2011) studies. In both studies, the perceived intensity of a standard tone was estimated through a volume comparison task between the standard tone and tones of differing intensities. Then the perceived intensity of the standard tone was compared between different conditions, e.g., between the self-generated tone and the computer generated tone. The same method will be used in the current study, with participants (Chinese and British) from both cultural backgrounds being tested in the same experimental setting to reduce any possible confounds. We also included a battery of questionnaires, including Self Construal Scale (Singelis, 1994), short forms of Empathy Quotient (EQ-short), short forms of Systemizing Quotient (SQ-short) (Wakabayashi et al., 2006) and Peters et al. (2004) Delusion Inventory (PDI), to measure cultural differences and cognitive style (for more information, please see Materials and Methods). Relevant to the focus of this study, we predict a correlation between sensory attenuation for others and self-construal, as self-construal plays an important role in cultural differences as outlined earlier.

## Materials and Methods

### Participants

Thirty Chinese (15 females, mean age = 22.4, *SD* = 1.7, 1 left-handed) and 30 British (15 females, mean age = 21.9, *SD* = 1.7, all right handed) participants, most of whom are students from the University of Glasgow, were recruited through a local subject pool. Chinese participants were all born and educated in China and they were tested within 3 months of their first arrival in UK. British participants were selected if their self-reported nationality information was UK and ethnicity information was British and White. All participants have self-reported normal hearing and normal or corrected to normal vision. Participants were debriefed and received a payment of £6/h after the experiment.

### Stimuli, Task and Procedure

Participants completed a sound comparison task between a standard tone and a comparison tone (i.e., which tone was louder). The standard tone was 74 dB sound pressure level in intensity and the comparison tone ranges from 71 to 77 dB with 1 dB increment. All tones (1000 Hz, 100 ms in duration, 10 ms rise/fall ramp, sampling rate at 48000 Hz) were generated with MATLAB (http://www.mathworks.com).

The experiment consisted of a pretesting phase and a testing phase. In the pretesting phase, participants pressed a button (number ‘2’ on numeric section of a standard keyboard) with their right index finger about once every 3 s. After a button press, they heard a standard tone immediately and received visual feedback on the screen whether the response was good, too slow (more than 3.8 s after last response) or too fast (less than 2.2 s after last response). There were 200 trials in total, and participants were allowed to take a break when needed. The purpose of including this pretesting phase was to keep the procedure the same as the procedure used in Sato (2008). Since the procedure was identical for participants from both cultural groups, the pre-testing phase was not crucial to the cultural difference question we were interested in.

**FIGURE 1:**
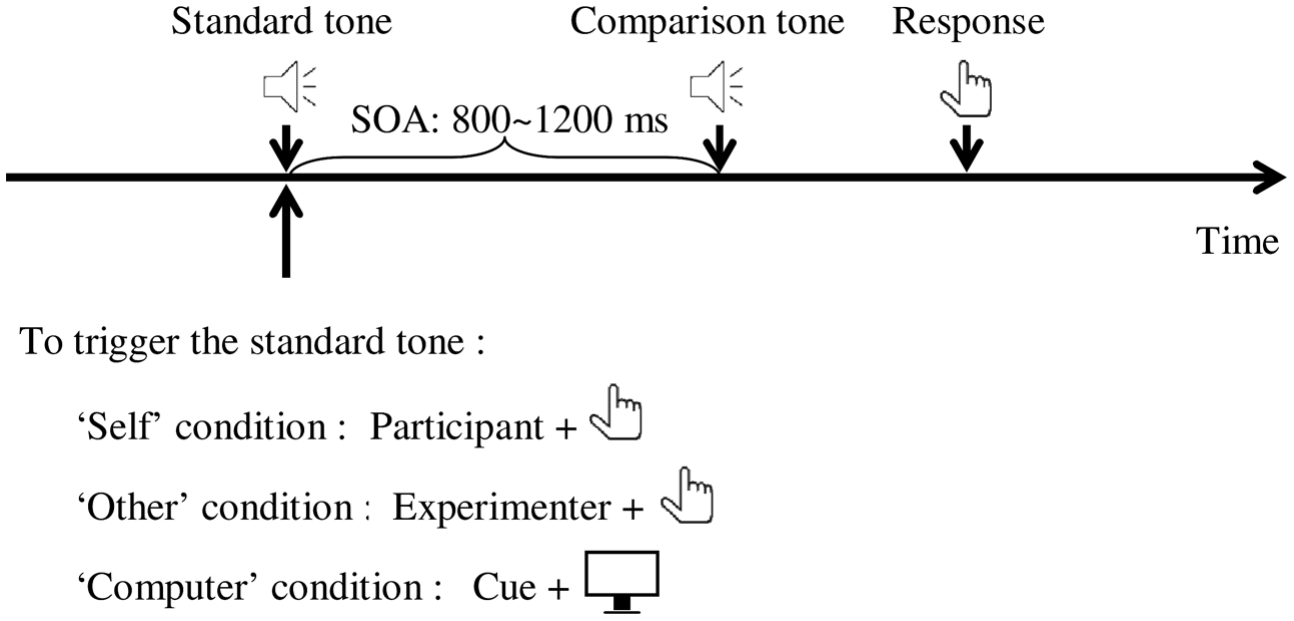
**Schematic illustration of a typical trial.** The standard tone was triggered by participant (‘self’ condition), experimenter (‘other’ condition), or computer (‘computer’ condition). The comparison tone played automatically after an SOA of 800–1200 ms. Participants were instructed to respond as accurately as possible which tone was louder. SOA, stimulus onset asynchrony.

In the testing phase (**Figure 1**), participants completed the sound comparison task. In each trial, participants first heard the standard tone. After a jittered interval of 800–1200 ms, they heard a comparison tone and then made a judgment which tone was louder by pressing button ‘F’ (if the first one is louder) or ‘J’ (if the second one is louder) on the keyboard with their left hand. The intensity of comparison tones was randomized across trials. There were three conditions in the testing phase that differed in the way the standard tone was triggered. In ‘self’ condition, the standard tone followed immediately after participants pressing the button ‘2’ with their right hand as in the pretesting phase. They were asked to press the button about once every 3 s after a response was made for the previous trial. No feedback of press latency was provided. In ‘other’ condition, the standard tone was triggered by the experimenter pressing the button ‘2’ with right hand in the same way as participants did in ‘self’ condition. Participants were required to pay attention to the experimenter’s hand in the whole process. In ‘computer’ condition, the computer controlled the presentation of the standard tone and participants received visual cues (from 1 s before the onset of standard tone, the cross in the screen center gradually enlarged in size and then changed its color from black to red just before the tone presentation) before the tone played. The onset of the standard tone was between 2.5 and 3.5 s after participants’ response for the previous trial. The three conditions each contained 210 trials (30 × 7 comparison tones) and were presented in a random order. Each condition was presented in three mini blocks, each containing 70 trials, and participants were offered a break after each mini block.

Tones were delivered to subjects through a set of headphones (Beyerdynamic, DT770 PRO Headset-250 OHM). A male experimenter (LC, Chinese) tested all the male participants and two female experimenters (one Ukrainian and one Swedish) tested all the female participants. Under this design, half of the participants from either cultural group were tested by a same race experimenter and the other half were tested by an other race experimenter. If there is an effect from the race of the experimenter, both groups should be equally affected. During the experiment, the experimenter sat next to the participants while in front of the stimulus computer all the time. Participants were told to try their best to avoid any unnecessary movements. Except in the ‘other’ condition, participants were asked to always fixate on the cross in the center of the screen. Participants’ behavior was monitored online by the experimenter. Questionnaires were completed after the experiment. Chinese participants completed translated versions of Self-construal Scale (Wang et al., 2008), EQ-short and SQ-short questionnaires (translated and back-translated). The experiment took between 2 and 2.5 h for each participant.

### Questionnaires

The included questionnaires were: Self Construal Scale (Singelis, 1994), short forms of Empathy Quotient (EQ-short), short forms of Systemizing Quotient (SQ-short) (Wakabayashi et al., 2006) and Peters et al. (2004) Delusion Inventory (PDI). Self Construal Scale is intended to measure individual’s self-construal pattern, which is the key measurement of cultural difference in the study. An independent self-construal score and a dependent self-construal score was provided. EQ-short and SQ-short questionnaires are developed based on Empathizing–Systemizing theory (Baron-Cohen, 2002). They are intended to be used to measure the general cognitive style (more social or more systematic). Since social interaction is involved in sensory attenuation for others, we suspect that the way other’s behavior is cognized could be very important. So it is an interesting question to see whether sensory attenuation for others is related to EQ or SQ. PDI measures individual delusional state, which is related to sensory attenuation for the self (see Discussion). The correlation between PDI and sensory attenuation for the self has been reported (Teufel et al., 2010). We hypothesize a replication of this correlation between PDI and sensory attenuation for the self.

### Data Analysis

Point of subjective equality (PSE) was used to assess the subjective perceptual intensity of the standard tone. To compute PSE, the percentage of comparison tones perceived as louder than the standard tone was computed for each of the seven intensities of the comparison tone and for each participant and condition. Data were fitted with a logistic function using the maximum-likelihood method (Wichmann and Hill, 2001). PSE is defined as the intensity where participants respond 50% of times that the comparison tone is louder. Three data sets from the British sample were excluded due to deviant PSE values (more than 2.5 standard deviations away from the mean), resulting in 30 data sets with Chinese sample and 27 data sets with British sample for the final analysis. Since group size is unbalanced, linear mixed-effects model was used to test the interaction and main effects of PSE (Baayen et al., 2008; Barr, 2013). The effects of sensory attenuation for the self and others were analyzed separately. When analyzing sensory attenuation for others, different linear mixed-effects models were built comprising of main and/or interaction effects from the 2 (cultural groups) by 2 (‘others’ and ‘computer’ conditions) design. For example, when testing the interaction effect, the model that includes both interaction effect and main effects was compared to the model that only includes main effects. A *p*-value will be derived from this model comparison. All significant effects will be followed up by t-test of PSEs between conditions. Sensory attenuation for the self was analyzed in the same way but including ‘self’ and ‘computer’ conditions instead of ‘other’ and ‘computer’ conditions. Besides PSE values, participants’ responses toward the 74 dB comparison tone were analyzed in the same way to assess the sensory attenuation effect. This is the sensitive part of the task where the standard tone and the comparison tone are actually identical (some studies only used this part in their design, see Stenner et al., 2014b; Reznik et al., 2015). When the two tones are identical, sensory attenuation would lead to more responses for the comparison tone as louder. So we calculated the percentage of responding the comparison tone as louder in this case, and an increase of this percentage value would indicate sensory attenuation.

For the correlation analysis, we quantified sensory attenuation the self effect by subtracting the PSE value in ‘computer’ condition from the PSE value in ‘self’ condition (SA_self_); sensory attenuation for others is measured by subtracting the PSE value in ‘computer’ condition from the PSE value in ‘other’ condition (SA_other_). Then correlation analysis was performed between SA_self_/SA_other_ and the four questionnaire scores with an open source Matlab toolbox that uses robust correlation (Pernet et al., 2012). Pearson’s correlation was used. Whenever a significant correlation was found, it was then tested with percentage-bend correlation and skipped-correlation for robust correlation tests. Pearson percentage-bend correlation results were reported in the main text.

### Ethics Statement

The experiment was conducted in accordance with ethical codes of conduct of APA, BPS, and declaration of Helsinki and was approved by the Ethics Committee of College of Science and Engineering, University of Glasgow. For each participant written consent was obtained prior to experiment.

## Results

### Sensory Attenuation for Others

**FIGURE 2:**
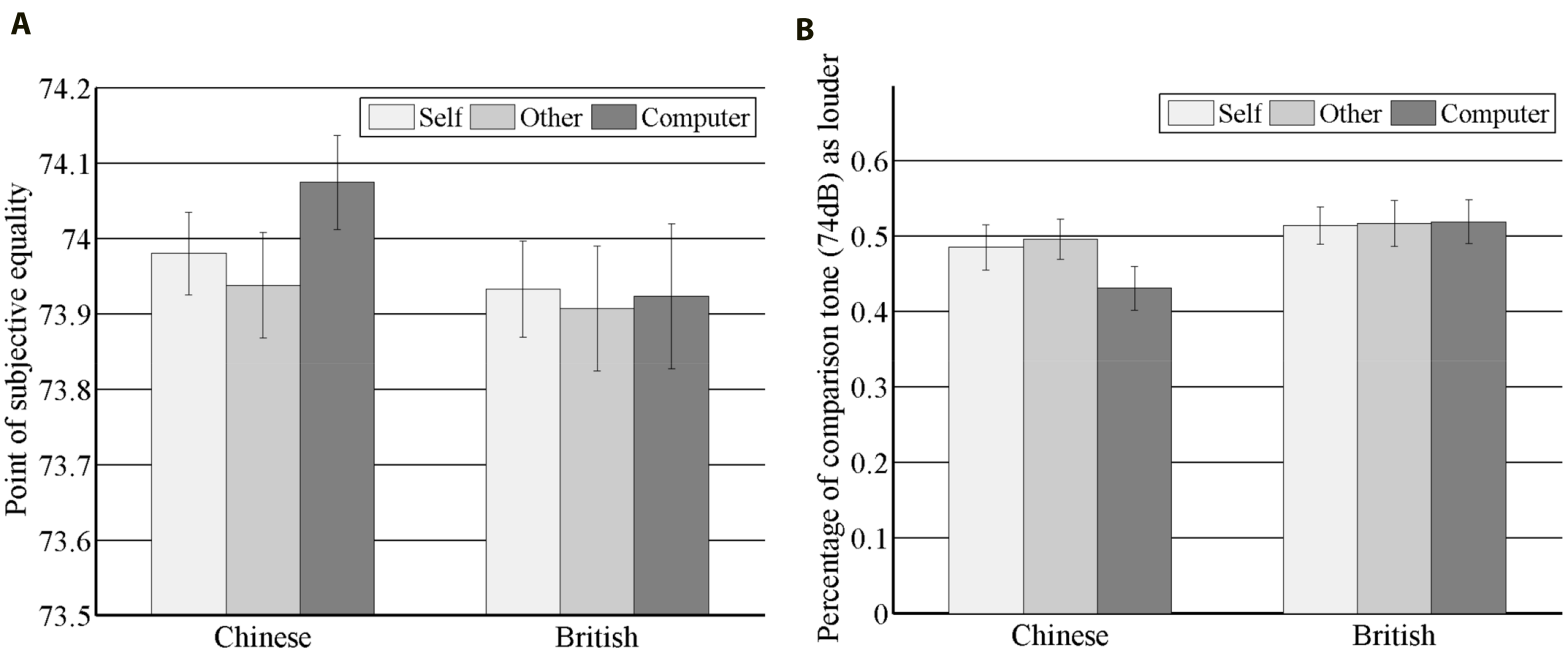
**Main results.** Point of subjective equality **(A)** and Percentage of comparison tone (74 dB) as louder **(B)** in three conditions for Chinese and British. Vertical bars represent standard error.

A significant main effect was found for conditions [χ^2^(1) = 4.11, *p* < 0.05], with PSE values smaller in ‘other’ condition (mean = 73.92; *SD* = 0.40) than in ‘computer’ condition (mean = 74.0; *SD* = 0.43) (**Figure 2A**). No other effects were significant [cultural groups: χ^2^(1) = 0.80, *p* = 0.37; interaction: χ^2^(1) = 2.58, *p* = 0.11]. Based on our a priori hypothesis about the cultural difference, we performed within cultural group *t*-test as a follow-up. The within cultural group *t*-test showed that sensory attenuation for others among Chinese was significant with a smaller PSE value in ‘other’ condition (*t*(29) = -2.79, *p* < 0.01, 95% Confidence Interval (CI) = [-0.24, -0.04], Cohen’s *d* = -0.51) and no such effect was found among British (*t*(26) = -0.28, *p* = 0.78, CI = [-0.13, 0.10], Cohen’s *d* = -0.05). Analysis with participants’ responses toward the 74 dB comparison tone resulted in a main effect for conditions [χ2(1) = 4.28, *p* < 0.05], i.e., more responses for the comparison tone as louder in ‘other’ conditions as compared to ‘computer’ condition. And most importantly, a significant interaction effect emerged [χ^2^(1) = 4.95, *p* < 0.05] (**Figure 2B**). *Post hoc* analysis gave similar results to the analysis with PSE. Chinese showed significant sensory attenuation for others with a higher percentage of responding comparison tone as louder in ‘other’ condition (mean = 0.50, *SD* = 0.15) than in ‘computer’ condition (mean = 0.43, *SD* = 0.16) (*t*(29) = 3.20, *p* < 0.01, CI = [0.02, 0.11], Cohen’s *d* = 0.58**)**, which was not the case for British participants (‘other’ conditions: mean = 0.52, *SD* = 0.16; ‘computer’ condition: mean = 0.52, *SD* = 0.15; *t*(26) = -0.11, *p* = 0.91, CI = [-0.05, 0.04], Cohen’s *d* = -0.02).

### Sensory Attenuation for the Self

Surprisingly, no main effects [cultural groups: χ^2^(1) = 1.31, *p* = 0.25; conditions: χ^2^(1) = 0.85, *p* = *0*.36] or interaction [χ^2^(1) = 1.29, *p* = 0.26] were found (**Figure 2A**). Following up within group *t*-test showed that there was a trending significant sensory attenuation for the self among Chinese with a lower PSE in ‘self’ condition (mean = 73.98, *SD* = 0.30) than in ‘computer’ condition (mean = 74.08, *SD* = 0.34) (*t*(29) = -1.86, *p* = 0.07, CI = [-0.20, 0.01], Cohen’s *d* = -0.34), and no sign of sensory attenuation for the self among British (‘self’ conditions: mean = 73.93, *SD* = 0.33; ‘computer’ condition: mean = 73.92, *SD* = 0.50; *t*(26) = 0.12, *p* = 0.90, CI = [-0.15, 0.17], Cohen’s *d* = 0.02). Analysis with responses for 74 dB comparison tone gave similar results (for Chinese: *t*(29) = 1.88, *p* = 0.07, CI = [-0.005, 0.11], Cohen’s *d* = 0.34; for British: *t*(26) = -0.19, *p* = 0.85, CI = [-0.06, 0.05], Cohen’s *d* = -0.04; no interaction effect: χ^2^(1) = 2.29, *p* = 0.13) (**Figure 2B**).

### Correlations

**FIGURE 3:**
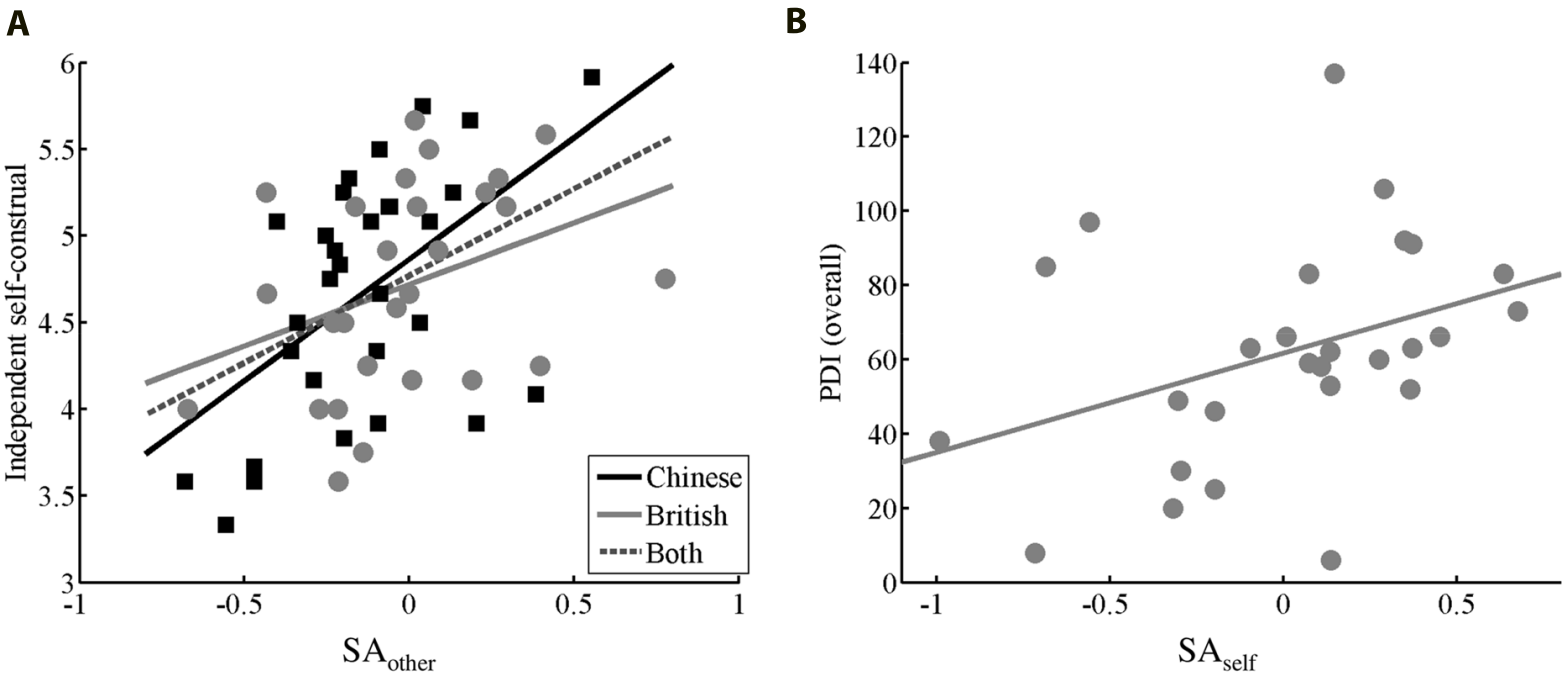
**Correlation results.** Scatter plot with SA_other_ on x-axis and Independent self-construal on y-axis **(A)**, and SA_self_ on x-axis and PDI (overall) score on y-axis **(B)**. Linear fit was shown with lines. Low SA_self_/SA_other_ values correspond to a strong sensory attenuation effect. PDI, Peters et al. (2004) delusion inventory; SA_self_, sensory attenuation for self-generated stimuli; SA_other_, sensory attenuation for others generated stimuli.

**Table 1** showed a summary of the correlations between SA_self_/SA_other_ and questionnaire scores. SA_self_ is the PSE difference between ‘computer’ condition and ‘self’ condition, which is used to quantify sensory attenuation for the self effect. And SA_other_ is used to quantify sensory attenuation for others effect (see Materials and Methods). For SA_other_, a significant correlation was found only with independent self-construal score (*r* = 0.40, *p* < 0.01, CI = [0.14, 0.65]; **Figure 3A**). Since negative values indicated positive SA_other_ effects, the correlation suggested that a higher independent self-construal score was associated with a reduced SA_other_. The correlation is still significant within the Chinese sample (*r* = 0.48, *p* < 0.01, CI = [0.03, 0.80]), and the British sample (*r* = 0.41, *p* < 0.05, CI = [0.02, 0.74]). For SA_self_, a significant correlation was found with PDI overall score among the British group (*r* = 0.44, *p* < 0.05, CI = [0.02, 0.76]; **Figure 3B**) but not among the Chinese group (*r* = -0.11, *p* = 0.56, CI = [-0.49, 0.24]). Among the British group, higher PDI overall scores (delusions) are associated with reduced SA_self_. SA_self_ also has promising correlation with other PDI break-down measurements: yes/no score (*r* = 0.39, *p* = 0.04, CI = [-0.04, 0.72]), distress score (*r* = 0.38, *p* = 0.05, CI = [-0.07, 0.71]), preoccupation score (*r* = 0.44, *p* = 0.02, CI = [0.02, 0.76]), conviction score (*r* = 0.40, *p* = 0.04, CI = [0.01, 0.73]). All significant (or trending significant) results remained significant when tested with normal Pearson correlation or other robust statistics such as skipped-correlation (see **Table 1** for results with normal Pearson correlation test).

**Table 1:** Summary of correlation analysis between SA_self_/SA_other_ and four questionnaires scores using Pearson’scorrelation and Pearson bend correlation.

Measure		EQ	SQ	Independent SC	Dependent SC	PDI (overall) (British only)
SA_self_	(*Method*) Pearson	-0.11	0.01	0.17	0	0.37^†^
		[-0.36, 0.14]	[-0.26, 0.25]	[-0.07, 0.40]	[-0.26, 0.30]	[0.02, 0.67]
	Pearson bend	-0.07	0	0.17	0	0.44^∗^
		[-0.35, 0.20]	[-0.28, 0.28]	[-0.12, 0.43]	[-0.29, 0.28]	[0.02, 0.76]
SA_other_	Pearson	0.01	0	0.44^∗^	-0.02	0.20
		[-0.27, 0.30]	[-0.28, 0.23]	[0.21, 0.64]	[-0.31, 0.31]	[-0.23, 0.65]
	Pearson bend	0.07	-0.04	0.40^∗^	0.06	0.15
		[-0.22, 0.38]	[-0.33, 0.23]	[0.14, 0.65]	[-0.23, 0.33]	[-0.28, 0.59]


*Data shown are Pearson’s r values, and 95% confidence intervals are included in the brackets. Pearson skipped correlations gave similar results. Note that the p-value for the correlation between SA_self_ and PDI overall is just above.05, but the bootstrapped 95% confidence interval is above 0 and Pearson bend correlation was significant. SA_self_, sensory attenuation for self-generated stimuli; SA_other_, sensory attenuation for others generated stimuli; EQ, empathy quotient; SQ, systemizing quotient; SC, self-construal; PDI, Peters et al. (2004) delusion inventory.*

*† p = 0.06; ^∗^p < 0.05.*

## Discussion

In this study, we report a cultural difference in perception of sensory consequences generated by others. Chinese participants showed sensory attenuation to stimuli generated by others, but this effect was not observed in British participants. Moreover, SA_other_ (quantifying sensory attenuation for others effect) was significantly correlated with independent self-construal, not interdependent self-construal or emphasizing abilities. Higher independent self-construal scores were related to smaller sensory attenuation for others effects. We failed to replicate sensory attenuation for the self in either Chinese or British sample, though a trend toward significance was found among Chinese (*p* = 0.07). However, a significant correlation between SA_self_ (quantifying sensory attenuation for the self effect) and delusional ideation was found among British.

Sensory attenuation for the self is generally considered as a consequence of internal forward model, which posits that along with any action an efference copy is sent to sensory areas that allows prediction of the expected sensory consequence of an action. Sensory attenuation for the self occurs when the reafferent signal corresponds to the expectation (Sperry, 1950; von Holst and Mittelstaedt, 1950; Wolpert and Ghahramani, 2000). Its functional role was suggested to help individuals distinguish self-generated stimuli from external stimuli, thus keeping the sense of agency (Blakemore et al., 1999; Sato and Yasuda, 2005). Schizophrenic patients suffer from impaired sense of agency and indeed they show reduced or diminished sensory attenuation for the self (Shergill et al., 2005; Ford et al., 2007). Specifically, the key factor responsible for the abnormality with sensory attenuation for the self was found to be delusional ideation (Heinks-Maldonado et al., 2007; Teufel et al., 2010). Supporting this, we found a significant correlation between SA_self_ measurement and delusional ideation among healthy subjects in the British sample (cf. Teufel et al., 2010). A closer examination of the correlation suggests that SA_self_ is related to almost all the aspects of delusional ideation as measured by the PDI break-down scores. Yes/no score (indicating the scope of delusional ideation) and distress score (indicating how distressing one feel about the delusional ideation) were either marginally significantly correlated with SA_self_ or the confidence interval for the correlation just contained 0. The correlations between SA_self_ and conviction score (indicating to what extent one is convinced of the delusional ideation), between SA_self_ and preoccupation score (indicating how much time one would spend on delusional ideation) were significant and had good confidence intervals suggesting that sensory attenuation for the self is more related to the intensity of delusional ideation than the scope or emotional consequences of delusional ideation. However, the correlation between SA_self_ and delusional ideation was not significant in the Chinese sample. We suspect that this may be because the PDI questionnaire is not suitable for Chinese population. This is evidenced by the surprisingly high PDI overall scores in our Chinese sample (mean: 102; median: 103). Peters et al. (2004) reported that in Western cultural context the mean scores for normal and deluded groups are 59 and 131, respectively. Since no norms are available for Chinese PDI scores, further studies are needed to clarify this question.

For unknown reasons we failed to obtain a significant sensory attenuation for the self effect, which has been previously shown (Sato, 2008; Weiss et al., 2011). Like previous studies, the standard tone in the current study was a unisensory stimulus in all conditions. The difference among conditions was what happened before the presentation of the tone. The ‘computer’ condition functioned to exclude the alternative explanation that the predictability of the stimulus onset explained the conditional differences. Similar manipulations for the control condition have been reported before (e.g., Ford et al., 2007). Sensory attenuation for the self renders a 74 dB tone to be perceived about 0.4 dB (Sato, 2008) or 0.2 dB (Weiss et al., 2011) softer, which is a very small perceptual effect and may be difficult to be detected. In our study, the 74 dB tone was perceived as 0.1 dB softer and 0.01 dB louder when it was self-generated for Chinese and British participants, respectively. Whereas the mean of SA_self_ may shift due to unknown reasons, the variance of SA_self_ across participants may not. This might be the reason why we can still find a significant correlation between SA_self_ (but not SA_other_) and delusional ideation, which also argues for the validity of the data. It is also worth noting that it’s unlikely that there is a cultural difference in sensory attenuation for the self, despite the numerical differences in SA_self_ found here. This is because that consistent cross-cultural results were reported on this topic (Sato, 2008; Weiss et al., 2011) and that the underlying theoretical forward model account applies universally (for example, it’s true that self-tickling is less ticklish than being tickled by others for both easterners and westerners; Crapse and Sommer, 2008). However, this should be left as an open question for future studies that have more robust measurements of sensory attenuation for the self. Unlike the behavioral measurement of sensory attenuation for the self, the electrophysiological measurement of this effect is very robust (Hughes et al., 2013; Schröger et al., 2015). Typically sound evoked responses are smaller when the sound is self-generated (e.g., ‘self’ condition in the current study) as compared to when it is from external sources (e.g., ‘computer’ condition in the current study). However, the relationship between the suppression of evoked responses and behavioral measurements of sensory attenuation is not clear from the literature. Some recent studies from the visual domain suggest that both effects may reflect the consequences of a common underlying mechanism, i.e., internal forward model (Stenner et al., 2014a; Hughes, 2015). In the auditory domain, we speculate that the suppression of evoked responses is a more unambiguous and direct measure of sensory attenuation but that the perceptual intensity judgment changes are a likely consequence of the alleged underlying mechanism. Further studies are needed to clarify this point.

Interestingly, Chinese but not British showed sensory attenuation for tones generated by others. This is consistent with our prediction stemming from the self-construal difference between cultural groups. The dominance of interdependency over independency in Eastern culture may lead to fewer differences between sensory consequences generated by others and self, thus easterners show sensory attenuation for others just like sensory attenuation for the self as reported in the literature. For westerners, stronger independency may make the sensory consequences from others distinct from sensory consequences from the self, thus the sensory consequences from others may be of no difference to external sensory stimuli. So westerners didn’t show sensory attenuation for sounds caused by others. Further support for the above explanation comes from the strong correlation between SA_other_ and the independent self-construal score. A large SA_other_ (large sensory attenuation for others effect) is associated with a small independent self-construal score. This reconciles the discrepant findings reported by Sato (2008) and Weiss et al. (2011). In the study by Sato (2008), participants and experimenter always pressed the same button with the same finger to trigger the same tone, whereas in the study by Weiss et al. (2011), participants and experimenter pressed different buttons to trigger a different (experiment 1) or same (experiment 2) tone. It is possible that the similarity between participants’ and experimenter’s response pattern leads to similar sensory attenuation effect following self and other’s movement in Sato (2008). By testing both groups of participants in the very same experimental setting, this possibility was ruled out and we confirmed sensory attenuation for others as a cultural phenomenon. A recent ERP study showing attenuated N1 neural responses to a tone after watching a goal-directed button press video was reported from Australia, where individualism is more prominent (Poonian et al., 2015). This seems to be at odds with Weiss et al. (2011) study and our data here. However, a different behavioral task (time estimation) in their study may hinder a direct comparison of the results. As discussed earlier, this may also suggest that sensory attenuation measured from cortical responses and perceptual intensity judgments may not exactly be the same thing.

Social context can modulate sensory processing (Desantis et al., 2012; Baess and Prinz, 2015). For example, Desantis et al. (2012) showed that sensory attenuation effect can be modulated by authorship belief. We also found that SA_other_ was significantly correlated with independent self-construal both when analyzing the two cultural groups together and separately, which suggests an influence of social orientation (general belief) on sensory processing. This is also direct evidence supporting the social orientation hypothesis on the origin of cultural differences in cognition (Varnum et al., 2010). According to the social orientation hypothesis, the fact that westerners are more independent and easterners are more interdependent is the origin to the various aspects of cultural differences in cognition. Independent/Interdependent self-construal is a key factor of the independency/Interdependency. SA_other_ is not correlated with EQ or SQ (see **Table 1**), which might suggest that empathizing-systemizing cognitive style is not related to sensory attenuation for others. What are the potential neural mechanisms underlying the sensory attenuation for others effect? One possible mechanism could be that other brain areas (possibly prefrontal cortex) modulate the neural responses in auditory cortex during this process (Müller et al., 2014). Another mechanism could be that the internal forward model still accounts for sensory attenuation for others, but it is activated by seeing other’s movement (Kilner et al., 2007; Poonian et al., 2015). As discussed by Sato (2008) and Weiss et al. (2011), mirror neurons could be the mediator. However, that would also assume a cultural difference in mirror neurons, which is under debate (Cook et al., 2014).

Given that Chinese showed sensory attenuation for others and British did not, and the effect was correlated with independent self-construal, it is reasonable to predict that a difference in independent self-construal would be found between the two groups. However, this is not the case (mean for Chinese: 4.67; mean for British: 4.71; *t*(55) = -0.20, *p* = 0.84, CI = [-0.38, 0.32]). The sample size could be too small to identify this difference. Another explanation is that the explicit self-construal measure in the Singelis self-construal scale is prone to situational influence (Cross et al., 2011). All the Chinese participants were tested shortly (mostly within 1 month) after their first arrival in UK, when they were trying to adapt to a new environment by themselves. That could promote their explicit sense of independence, which was reflected in Singelis self-construal scores. Independence as revealed through sensory attenuation for others can be more resistant to the influence from social environment within a short period of time, as it is more like an implicit measure of attitudes toward others. Whether or not it changes with more enculturation is an interesting question for further studies. Contrary to independent self-construal, we found a significant difference in interdependent self-construal with Chinese scoring higher (mean for Chinese: 5.25; mean for British: 4.72; *t*(55) = 2.87, *p* < 0.01, CI = [0.16, 0.90]). Interestingly, interdependent self-construal was not correlated with SA_other_ (cf. Kitayama and Park, 2014). We view this as supporting evidence that independent and interdependent self-construals are two non-reducible aspects of self (Singelis, 1994) and may have independent influence on cognition and behavior. For example, Kitayama and Park (2014) showed that the culture difference in self-centric motivation was mediated by interdependent self-construal but not independent self-construal.

## Conclusion

There is a profound cultural difference in processing sensory consequences generated by others, with people from collectivism-dominated culture backgrounds showing sensory attenuation and people from individualism-dominated culture backgrounds not. Sensory attenuation in this case is related to independent self-construal; however, it may operate in a deeper layer thus being a more reliable measurement of one’s social orientation. Further studies should address its development and cognitive mechanisms (especially its relationship to sensory attenuation for the self).

## Author Contributions

LC and JG developed the study concept and designed the study. Data collection and analysis were performed by LC. LC and JG interpreted the data. LC drafted the manuscript, and JG provided critical revisions. Both authors approved the final version of the manuscript for submission.

## Data Availability Statement

The original data are available from: Harvard Dataverse http://dx.doi.org/10.7910/DVN/GQPCII

## Conflict of Interest Statement

The authors declare that the research was conducted in the absence of any commercial or financial relationships that could be construed as a potential conflict of interest.
